# Lysosomes as Oxidative Targets for Cancer Therapy

**DOI:** 10.1155/2017/3749157

**Published:** 2017-07-05

**Authors:** Rebecca F. Dielschneider, Elizabeth S. Henson, Spencer B. Gibson

**Affiliations:** ^1^Providence University College, Otterburne, MB, Canada; ^2^Research Institute in Oncology and Hematology, CancerCare Manitoba, 675 McDermot Ave., Winnipeg, MB, Canada; ^3^Department of Biochemistry and Medical Genetics, Faculty of Health Sciences, University of Manitoba, Winnipeg, MB, Canada

## Abstract

Lysosomes are membrane-bound vesicles that contain hydrolases for the degradation and recycling of essential nutrients to maintain homeostasis within cells. Cancer cells have increased lysosomal function to proliferate, metabolize, and adapt to stressful environments. This has made cancer cells susceptible to lysosomal membrane permeabilization (LMP). There are many factors that mediate LMP such as Bcl-2 family member, p53; sphingosine; and oxidative stress which are often altered in cancer. Upon lysosomal disruption, reactive oxygen species (ROS) levels increase leading to lipid peroxidation, mitochondrial dysfunction, autophagy, and reactive iron. Cathepsins are also released causing degradation of macromolecules and cellular structures. This ultimately kills the cancer cell through different types of cell death (apoptosis, autosis, or ferroptosis). In this review, we will explore the contributions lysosomes play in inducing cell death, how this is regulated by ROS in cancer, and how lysosomotropic agents might be utilized to treat cancers.

## 1. Introduction

Lysosomes are membrane-enclosed vesicles that contain at least 60 hydrolases within an acidic environment. These hydrolases, which include the cathepsin family of proteases, are responsible for degradation, recycling, and disposal of cellular macromolecules [1]. Lysosomes are often termed the garbage disposal of the cell, but as our knowledge and understanding increase, the roles lysosomes play in other cellular functions expand [2]. The lysosomal degradation pathway regulates a variety of cellular functions such as autophagy, endocytosis, and phagocytosis to maintain cellular homeostasis [1]. In addition, this pathway directly or indirectly regulates cell signaling, metabolism, and degradation of protein aggregates and damaged organelles [3–5]. When the degradative pathway is dysregulated, diseases such as cancer can progress. This makes lysosomes a potential target for cancer therapy.

## 2. Lysosomal Biology

Lysosomes are the most acidic vesicles within the cell. This acidic pH is maintained by the action of a proton pump which hydrolyzes ATP to ADP in order to pump an H^+^ ion into the lumen of the lysosome [6]. The lysosomal membrane consists of a lipid bilayer and membrane proteins. The most abundant lysosomal membrane proteins are lysosome-associated membrane proteins 1 and 2 (LAMP-1 and LAMP-2). The inner lumen of these proteins is highly glycosylated and protects the lysosomal membrane from the digestive enzymes [7, 8]. These enzymes can digest DNA, RNA, sugars, lipids, and proteins. Among these enzymes is the diverse cathepsin protease family. Cathepsins A and G are serine proteases, meaning that their active site contains a vital serine. Cathepsins B, C, F, H, K, L, O, S, V, X, and W are cysteine proteases. Cathepsins D and E are aspartic proteases. Cysteine cathepsins are the most stable and active at an acidic pH. Like caspases, cathepsins have a wide range of cellular substrates. Cystatins, thyropins, and serpins prevent cathepsin substrates from binding and are thus endogenous inhibitors of cathepsins [9].

Lysosomal biogenesis is controlled by master regulators transcription factor EB (TFEB) and microphthalmia-associated transcription factor (MITF). These proteins receive cues in the cytoplasm and translocate into the nucleus to induce the transcription of lysosomal biogenesis network of genes [5, 10, 11]. TFEB and MITF are phosphorylated by mTOR in the cytoplasm and retained there by binding to 14-3-3 proteins [10]. Upon inhibition of the mTOR pathway under stress conditions, lysosomal biogenesis could be activated.

## 3. Lysosomes in Cancer

Lysosomes have been associated with diseases such as lysosomal storage disorders, neurodegenerative disorders, and cardiovascular disease [12, 13]. In cancer, lysosomal function is also altered. Many cancer cells have increased the number of lysosomes to maintain homeostasis by the increased degradation and recycling macromolecules to maintain cell proliferation and survive under stress condition in the microenvironment [4, 14, 15]. Indeed, increased expression of cathepsin B has been associated with increased cancer invasion [16]. Despite the ubiquitous nature of lysosomes in all mammalian cell types, cancer cells have been shown to increase lysosomal biogenesis [14, 17] and alter cellular biology [18, 19], thus affecting lysosomes. One such biological process that impacts lysosomes is sphingolipid metabolism. Altered sphingolipid metabolism has been found in many cancers [20–22]. Different cancer cell types overexpress sphingosine kinase (SK) [23–25] and downregulate acidic sphingomyelinase (ASM) [19]. These changes affect lysosomal membrane structure and function in cancer cells.

Lysosomes also play an important role in drug resistance in cancer by sequestering weak-base chemotherapeutic drugs within the cell. This increases lysosomal biogenesis resulting in enlargement of the lysosomal compartment in cells [15]. The enlarged compartment allows significant concentration of chemotherapeutic drugs to be stored in lysosomes and blocks these drugs from reaching their cellular targets. In addition, lysosomes provide a mechanism for exocytosis of drugs from the cancer cells [15]. These mechanisms render cancer cells drug-resistant, thus highlighting lysosomes as a target for cancer therapy.

## 4. Lysosomal Membrane Permeabilization (Figure 1)

Lysosomal membrane permeabilization (LMP) has been shown to be an effective therapeutic strategy in many cancer models [26]. LMP involves either the slight or the complete permeabilization of the lysosome. This permeabilization can cause lipid peroxidation and a partial or complete release of lysosomal contents. Cell death can be mediated by the reactive oxygen species (ROS) and/or lysosomal cathepsins [3, 4, 26]. In addition, sphingolipids can contribute to LMP [27]. Sphingosine has been shown to induce LMP when added to cells [27]. Upon TNF*α*, radiation, and DNA-damaging drug treatments, p53 is phosphorylated and translocates to lysosomes where it induces LMP [5]. Various cellular components can protect the lysosome from permeabilization such as cholesterol [28], lysosomal localization of heat shock protein 70 [29], and lipid peroxidation scavengers. Tocopherols are endogenous inhibitors of lipid peroxidation. Among tocopherols is *α*-tocopherol, otherwise known as vitamin E [30, 31]. Thus, there are many factors regulating LMP in cancer cells.

Cancer cells are sensitive to LMP by a variety of mechanisms. Cell lines transformed with oncogenic Src and Ras display altered lysosomal localization and decrease in LAMP-1 and LAMP-2 [18]. Decreases in the LAMP proteins prime cells for LMP. Other cancer cells increase lysosomal biogenesis [14, 17], increase lysosomal size, and alter heat shock protein 70 (HSP-70) localization creating destabilized lysosomes [29]. Cancer cells have altered sphingolipid metabolism which increases the amount of sphingosine and renders lysosomes sensitive to LMP [22, 27, 32]. Finally, many cancer cells have altered metabolism that increases ROS leading to destabilization of lysosomes leading to LMP [3, 23]. Thus, cancer cells might be sensitive to lysosome-mediated cell death.

## 5. Lysosome-Mediated Cell Death (LCD)

Since their discovery as the suicide bags of the cell, lysosomes have been explored as therapeutic targets in cancer. Due to these numerous alterations to this pathway, LMP is an effective way to kill many different cancer cell types. These include breast cancer [19, 30, 33], ovarian cancer [19], cervical cancer [19], colon cancer [18, 34, 35], prostate cancer [19], lung cancer [35–37], bone cancer [19], skin cancer [35], and AML [14]. Cancer cells are susceptible to lysosome-mediated cell death through increased ROS and lipid peroxidation leading to mitochondrial dysfunction and plasma membrane permeabilization [38]. Furthermore, the release of cathepsins caused cleavage and degradation of proteins leading to cell death [3]. The relations of lysosome-mediated cell death with other forms of cell death will be discussed below.

## 6. Lysosomes and Apoptosis

Apoptosis is a form of program cell death involving mitochondrial dysfunction and activation of cysteine proteases called caspases. It leads to DNA condensation and membrane blebbing and eventually to the formation of apoptotic bodies that are phagocytosed by the surrounding cells. Mitochondrial dysfunction is triggered by the translocation of the Bcl-2 family member Bax to the mitochondria where it interacts with Bak and other BH3-only Bcl-2 family members such as Bid to form a pore allowing cytochrome c to be released and loss of membrane potential to occur. This leads to an increase in ROS and activation of caspase 9 and caspase 3 leading to cell death [39].

Lysosomes could play important roles in regulating apoptosis upstream of mitochondrial function and after caspase activation. Following oxidative stress, it was shown that low concentrations of hydrogen peroxide cause LMP before mitochondrial dysfunction and caspase activation [40]. Blocking cathepsin D activation also prevented the release of mitochondrial cytochrome c and caspase activation [41]. Moreover, ultraviolent radiation induces LMP under conditions of oxidative stress before mitochondrial release of cytochrome c [42]. Bax interacts with other BH3-only Bcl-2 family members such as BIM and BID at lysosomes contributing to LMP independent of its mitochondrial functions. BID is also a target of cathepsins allowing its translocation to the mitochondria to interact with Bax and Bak [42]. Similar to mitochondrial regulation, antiapoptotic Bcl-2 family members can prevent LMP [26]. This suggests that lysosomal disruption can lead to mitochondrial dysfunction.

Lysosomal disruption can also occur after mitochondrial dysfunction. Following loss of membrane potential, ROS production is increased. ROS destabilizes lysosomal membranes through lipid peroxidation leading to rupture [14, 27]. Activation of caspase 8 by death receptors or activation of caspase 9 has been associated with LMP [36, 43]. Overall lysosomes can play a role in either initiating or executing apoptosis.

## 7. Lysosome and Autophagy

Lysosomes fuse with autophagosomes forming an autolysosome to degrade extracellular or intracellular material [44]. Autophagy plays important roles in cancer cell adaptation to stress where it protects cancer cells from death during development and where its induction is limited to further progression of the disease [45]. Lysosomes function in autophagy regulation in three main areas: (i) lysosomal restoration, (ii) lysophagy, and (iii) autolysosomal degradation. Under normal conditions, lysosomal biogenesis occurs through biosynthesis and endocytic pathways to maintain homeostasis. Under stress conditions, the number of lysosomes decreased due to their role in degrading macromolecules for recycling or removing damaged organelles. Lysosomal levels are restored through a process called autophagic lysosomal reformation (ALR) [46]. This process can be prevented by autophagy inhibitors such as rapamycin and cathepsin inhibitors [46]. The second way autophagy regulates lysosomes is when lysosomes themselves become damaged such as through LMP. The damaged lysosomes are engulfed by autophagosomes which then fuse with functional lysosomes to remove them from the cells [47]. The levels of lysosomes are then restored by lysosomal biogenesis. Finally, the fusion of lysosomes and autophagosomes provides essential amino acids and nutrients to the cell and degrades damaged organelles [48]. If this process was left unchecked, the destruction of intracellular structures will lead to cellular collapse and a form of cell death called autosis [49]. This is dependent on functional lysosomes.

## 8. Lysosome and Ferroptosis

Ferroptotic cell death is a type of cell death that is distinct from apoptosis and autophagy [50, 51]. It is characterized by iron-dependent accumulation of ROS. Several proteins responsible for the regulation of iron such as ferritin and transferrin and the cysteine antiporter receptor have implicated the regulation of ferroptosis [52, 53]. One of the major storage sites for iron is lysosomes. In the presence of hydrogen peroxide, free iron undergoes a Fenton reaction creating reactive iron and increasing ROS [38]. The lysosomal disruptor siramesine induces a rapid rise in the lysosomal pH followed by lysosomal leakage mediated in part by inhibiting sphingomyelinase (ASM) [19]. This destabilization of lysosomal membranes leads to increased reactive iron and increased ROS causing cell death [30]. We found that the combination with a dual tyrosine kinase inhibitor of ErbB1 and ErbB2 tyrosine kinase receptors called lapatinib with siramesine could induce ferroptosis through blocking iron transport allowing the iron released by lysosomal disruption to accumulate and increase ROS [54]. The role lysosomes play in regulating ferroptosis through increased active iron and ROS requires future investigations.

## 9. Lysosomotropic Agents

LMP can be induced by numerous different stimuli that are collectively called lysosomotropic agents. Lysosomotropic agents are weak-base lipophilic or cationic amphiphilic drugs that accumulate in lysosomes. This occurs through diffusion across the lysosomal membrane where the agents become protonated and become trapped in the lysosome [26]. This causes damage to the lysosomal membranes leading to LMP. Lysosomotropic agents include metal nanoparticles [55], kinase inhibitor ML-9 [56], and numerous different types of pharmaceuticals. Pharmaceutical lysosomotropic agents include the antidepressants siramesine, nortriptyline, desipramine, imipramine, and clomipramine [19]. These have shown effectiveness in breast cancer, colon cancer, and CLL cells. Antimalarials mefloquine and chloroquine have shown effectiveness in breast cancer, lymphoma, and leukemia cells [14, 57–59]. Chloroquine has been investigated in clinical trials with only partial activity in lymphoma reported. There is, however, no evidence in these trails that chloroquine is acting through LMP. Antiallergy drugs terfenadine and loratadine [19] were effective at inducing cell death in breast and lung cancer cells. The treatments of stilbenoid antioxidant pterostilbene [35, 60] and antipsychotics chlorpromazine, thioridazine, and aripiprazole [19] showed efficacy in breast and leukemia cells. The use of these agents is summarized in Table 1. Many of these agents are FDA-approved or have been extensively studied in clinical trials but, with the exception of chloroquine, not in cancer patients [61]. This provides the foundation for many of these lysosomotropic agents to be clinically investigated for their efficacy in a variety of cancers in the near future.

## 10. Conclusion

Lysosomes play a dynamic role in cells and are altered in cancer. The initiation of LMP in cancer cells is a novel mechanism to engage the different cell death mechanisms selective for cancer. LMP is induced by lysosomotropic agents through increased ROS, lipid peroxidation, and activation of cathepsins. Many of these lysosomotropic agents are FDA-approved and could be moved rapidly to the clinic. Targeting lysosomes to induce oxidative stress will be dependent on the context of other therapies and drug resistance mechanisms found in cancer cells. Further investigation is needed to understand the regulation of lysosome-mediated cell death and the use of lysosomotropic agents in combination with other standard chemotherapy drugs or novel targeted anticancer drugs. Nevertheless, targeting lysosomes provides hope that effective treatment against drug-resistant cancers could be developed.

## Figures and Tables

**Figure 1:**
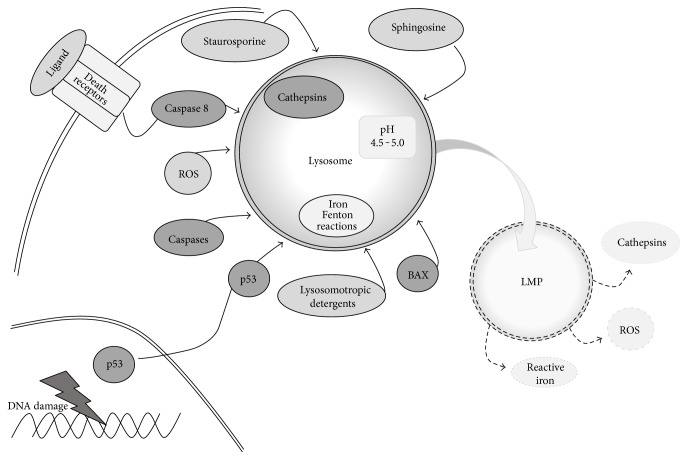
Regulation of lysosomal membrane permeabilization (LMP). There are many factors that regulate lysosomal membrane permeabilization (LMP). These include increased levels of sphingosine, cathepsins, and ROS. The activation of caspase, Bax, and p53 and treatment with staurosporine, or lysosomotropic agents, also lead to LMP. This results in the release of ROS, cathepsins, and reactive iron from lysosomes.

**Table 1:** The use of lysosomotropic agents as therapeutics in cancer.

Lysosomotropic Agent		Model	Effective doses	Reference
Siramesine	In vitro	Breast cancer lines: Mcf-7, Mcf-10A, and MDA-MB-468	1–10 *μ*M	[18, 19, 30]
		Cervix carcinoma cell lines: HeLa and ME-180	1–10 *μ*M	
		Colorectal cancer cell lines: Hkh2 and HCT116	8 *μ*M	
		Fibroblast cell line: NIH3&3-SrcY527F	4–10 *μ*M	
		Fibrosarcoma cell lines: WEHI-S and R4	5 *μ*M	
		Mast cells (primary)	2–20 *μ*M	
		Osteosarcoma cell line: U2OS	1–10 *μ*M	
		Ovarian carcinoma cell line: SKOV3	8–10 *μ*M	
		Prostate cancer cell lines: PC3 and Du145-P	5–10 *μ*M	
	In vivo	WEHI-R4 in BALB-c mice	25–100 mg/kg/d	
		Mcf-7 in SCID mice	30–100 mg/kg/d	
		PC3-MDR in SCID mice	30 mg/kg	

Desipramine	In vitro	Breast cancer lines: Mcf-7 and Mcf-10A	25 *μ*M	[19]
		Cervix carcinoma cell line: HeLa	25–50 *μ*M	
		Colorectal cancer cell lines: Hkh2 and HCT116	8 *μ*M	
		Fibroblast cell line: NIH3&3-SrcY527F	8–25 *μ*M	
		Osteosarcoma cell line: U2OS	25–50 *μ*M	
		Ovarian carcinoma cell line: SKOV3	75–100 *μ*M	
		Prostate cancer cell lines: PC3 and Du145-P	5–10 *μ*M	
	In vivo	Mcf-7 in SCID mice	30 mg/kg, 2×/wk	

Nortriptyline	In vitro	Breast cancer line: Mcf-7	25–50 *μ*M	[19]
		Cervix carcinoma cell line: HeLa	25–50 *μ*M	
		Colorectal cancer cell lines: Hkh2 and HCT116	8 *μ*M	
		Fibroblast cell line: NIH3&3-SrcY527F	10–25 *μ*M	
		Osteosarcoma cell line: U2OS	25–50 *μ*M	
		Ovarian carcinoma cell line: SKOV3	40–60 *μ*M	
		Prostate cancer cell lines: PC3 and Du145-P	40–80 *μ*M	

Amlodipine	In vitro	Breast cancer line: Mcf-7	25–50 *μ*M	[19]
		Fibroblast cell line: NIH3&3-SrcY527F	10–30 *μ*M	
		Ovarian carcinoma cell line: SKOV3	37.5–50 *μ*M	
		Prostate cancer cell lines: PC3 and Du145-P	40–50 *μ*M	

Terfenadine	In vitro	Breast cancer line: Mcf-7	25–50 *μ*M	[19]
		Colorectal cancer cell lines: Hkh2 and HCT116	8 *μ*M	
		Fibroblast cell line: NIH3&3-SrcY527F	2.5–5 *μ*M	
		Ovarian carcinoma cell line: SKOV3	6–8 *μ*M	
		Prostate cancer cell lines: PC3 and Du145-P	1–10 *μ*M	
	In vivo	Mcf-7 in SCID mice	10 mg/kg, 2×/wk	

Mefloquine	In vitro	AML cells (primary)	5–15 *μ*M	[14]
		AML cell lines: HL60, KG1A OCI-AML2, and TEX	1–10 *μ*M	
		APL cell line: NB4	5–7 *μ*M	
		CML cell line: K562	6–10 *μ*M	
		Dendritic cells (primary)	25–50 *μ*M	
		Erythroleukemic cell line: OCI-M2	7–9 *μ*M	
		Gastric cancer cell lines: AGS, Hs746T, MKN45, MKN74, NCI-N87, SNU1, SNU16, TCC1, YCC10, and YCC11	0.5–5 *μ*M	
		Lymphosarcoma cell line: MDAY-D2	3–5 *μ*M	
		Macrophage/monocyte cell lines: THP-1 and U937	5–18 *μ*M	
		Oral cancer cell line: KVP20C	5 *μ*M	
		Prostate cancer cell line: PC3	5–40 *μ*M	
	In vivo	K562, MDAY-D2, and OCI-AML2 in NOD-SCID mice	50 mg/kg	
		Primary AML cells in NOD-SCID mice	100 mg/kg/d	
		YCC or SNU1 in SCID mice	Unknown	
		PC3 in C57B1/6J mice	200 *μ*g/25 mg	

Primaquine	In vitro	Breast cancer cell line: Mcf-7	7 *μ*M	[58]
		Colon cancer cell lines: Caco-2 and HT-29	40–70 *μ*M	
		Oral cancer cell line: KVB20C	50–75 *μ*M	

Atovaquone	In vitro	Oral cancer cell line: KVB20C	2–12.5 *μ*M	[59]

Ciprofloxacin	In vitro	Cervix carcinoma cell line: HeLa	10 *μ*g/ml	[34]
		Colorectal cancer cell line HCT116	1–5 *μ*M	

Pterostilbene	In vitro	AML cell lines: HL-60, MV4-11, and OCI-AML2	25–75 *μ*M	[58]
		Macrophage cell lines: THP-1 and U937	25–75 *μ*M	
		Melanoma cell line: A375	10–50 *μ*M	
